# Refractory Dermatitis Evolving Into Lupus Spectrum Disease in the Setting of Dupilumab Use

**DOI:** 10.7759/cureus.79567

**Published:** 2025-02-24

**Authors:** Jonathan M Joseph, Jacob Kilgore, Nicholas Culotta

**Affiliations:** 1 Department of Dermatology, Louisiana State University (LSU) Health, New Orleans, USA

**Keywords:** autoimmune, cutaneous lupus erythematosus, dupilumab, hydroxychloroquine, lupus spectrum disease

## Abstract

Cutaneous lupus erythematosus (CLE) encompasses a broad range of clinical and histopathologic variants that can overlap with other dermatologic entities, complicating accurate diagnosis. We report the case of a 42-year-old male patient who initially presented with a diffuse pruritic eruption presumed to be atopic dermatitis, for which dupilumab was initiated. Within the following weeks, the patient developed a fever of unknown origin and diarrhea, raising concern for an atypical drug-related reaction or an unmasked autoimmune process. Subsequent biopsies demonstrated evolving histopathologic features, including superficial and deep perivascular dermatitis suggestive of drug eruption. In addition, a dermal mucin deposition with mixed neutrophilic and lymphocytic infiltrates is suggestive of cutaneous lupus, such as tumid lupus or lupus-related neutrophilic urticarial dermatosis.

Despite negative direct immunofluorescence and fluctuating autoantibodies, partial and sustained clinical improvement occurred with hydroxychloroquine therapy. The patient’s variable serologic profile (including intermittent positivity for antiribonucleoprotein and anti-Smith), transient urticarial lesions, and evolving histopathology highlight the difficulties in definitively categorizing cutaneous lupus subtypes. While a direct causal link between dupilumab and lupus-like disease remains unproven, the temporal association raises the possibility that T helper type 1/T helper type 2 immune modulation may unmask subclinical autoimmune conditions.

This case underscores the importance of repeated clinicopathologic correlation and multidisciplinary surveillance in patients presenting with atypical or treatment-refractory dermatitis. Ongoing dermatologic and rheumatologic evaluation is critical for early detection of systemic involvement, especially when autoimmune etiologies are suspected. Hydroxychloroquine remains a cornerstone of therapy for many CLE variants and can provide substantial improvement, even in complex or overlapping clinical scenarios.

## Introduction

Cutaneous lupus erythematosus (CLE) represents a broad spectrum of autoimmune skin disorders that can manifest with diverse morphologic features, varying severities, and distinct histopathologic findings [1,2]. Diagnosing CLE can be especially challenging due to overlapping clinical features with other inflammatory dermatoses, including drug eruptions, eczematous processes, and urticarial conditions. Furthermore, patients may exhibit incomplete or fluctuating serologic markers of systemic autoimmune activity, making it difficult to definitively categorize their disease [3,4]. Hydroxychloroquine is generally considered a first-line therapy for many forms of cutaneous and systemic lupus; however, the response can be partial or delayed, requiring ongoing clinical and histopathologic reassessment [5,6].

We present a case of a patient who was treated by an outside nondermatologic clinic with dupilumab for pruritus and a nondescript eruption initially diagnosed as atopic dermatitis. The patient subsequently developed cutaneous lupus, a phenomenon that may have been unmasked by dupilumab given the temporal relationship. Although a direct causal link cannot be definitively confirmed, existing case reports have documented lupus-like phenomena arising after initiating dupilumab, suggesting a possible immunologic shift, potentially T helper type 1 (Th1)-driven, that may unmask or exacerbate subclinical autoimmune conditions [7].

The proposed mechanism centers on dupilumab’s modulation of the immune response, particularly its inhibition of the Th2 pathway. As a monoclonal antibody targeting the IL-4 and IL-13 signaling pathways, dupilumab reduces Th2-mediated responses, which can inadvertently tip the immune balance toward Th1 and Th17 pathways. In the context of CLE, this shift may promote the pathogenesis of autoimmune diseases such as lupus erythematosus by enhancing Th1/Th17-driven inflammation [7-9]. Furthermore, dupilumab’s blockade of IL-4 and IL-13 may be ineffective, or even detrimental, in Th1-related disorders like systemic lupus erythematosus (SLE), potentially accelerating disease progression. Case reports indicating new-onset or worsening SLE after dupilumab initiation bolster the possibility that while dupilumab treats Th2-dominant conditions effectively, it may simultaneously unmask or worsen conditions underpinned by Th1/Th17 immunity [7].

## Case presentation

We present the case of a 42-year-old male patient who presented to the emergency department on September 25, 2023, with a pruritic eruption that initially appeared on his elbows and knees before progressively spreading to his arms, legs, trunk, and neck. There, he received a dexamethasone injection, oral prednisone, famotidine, and loratadine. He noted mild improvement; however, after discontinuing the corticosteroid, the eruption worsened. In early October 2023, he presented to the same outside provider, where he received a loading dose of dupilumab for presumed atopic or eczematous dermatitis, after which he developed a fever of unknown origin and diarrhea, necessitating hospitalization. While hospitalized, his cutaneous eruption had resolved except for an injection-site reaction, and an extensive workup by oncology and rheumatology did not reveal malignancy or a definitive, primary rheumatologic disease.

**Figure 1:**
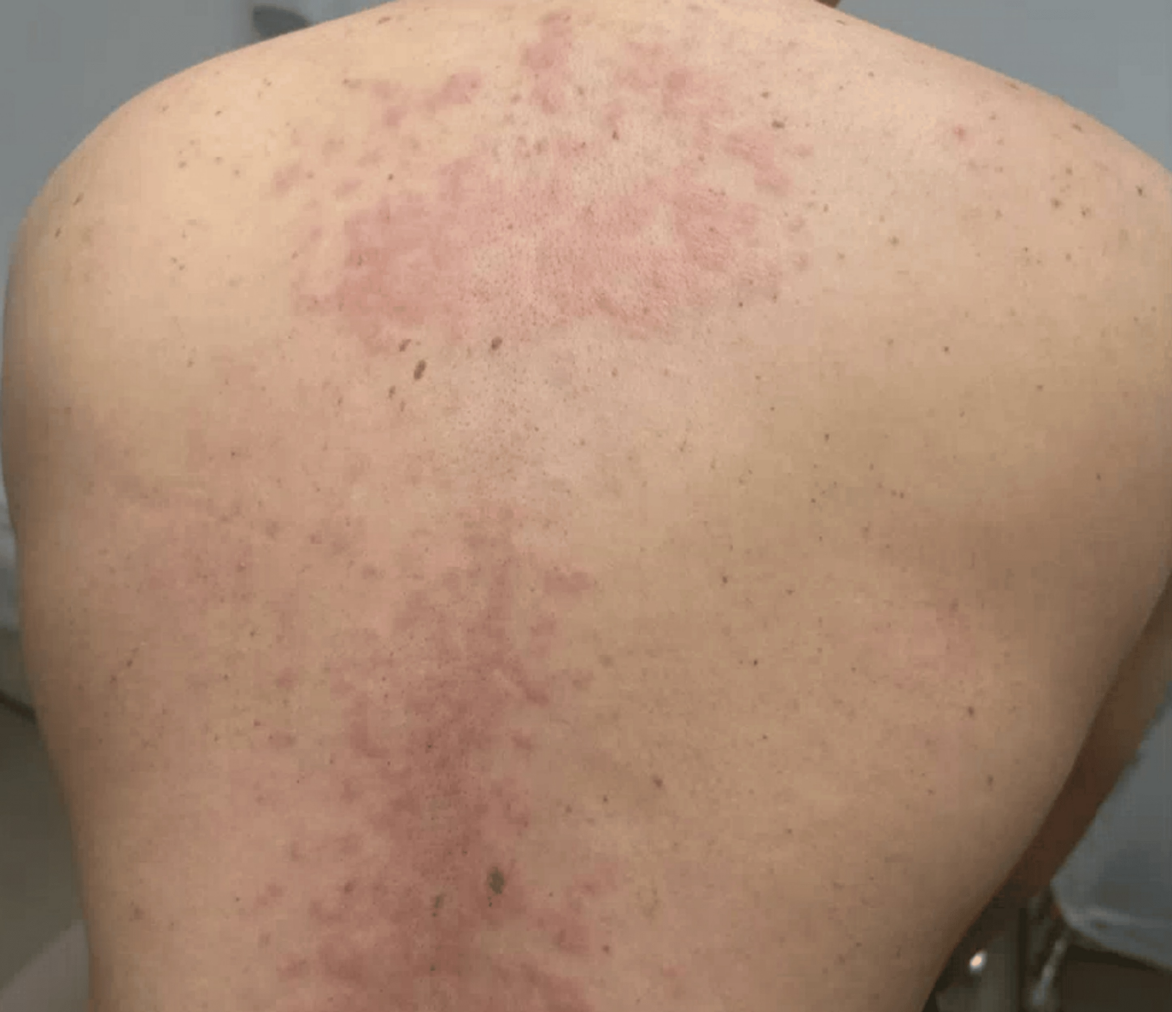
Recurrence of cutaneous eruption following discontinuation of topical corticosteroids The patient's back with erythematous papules coalescing into plaques

**Figure 2:**
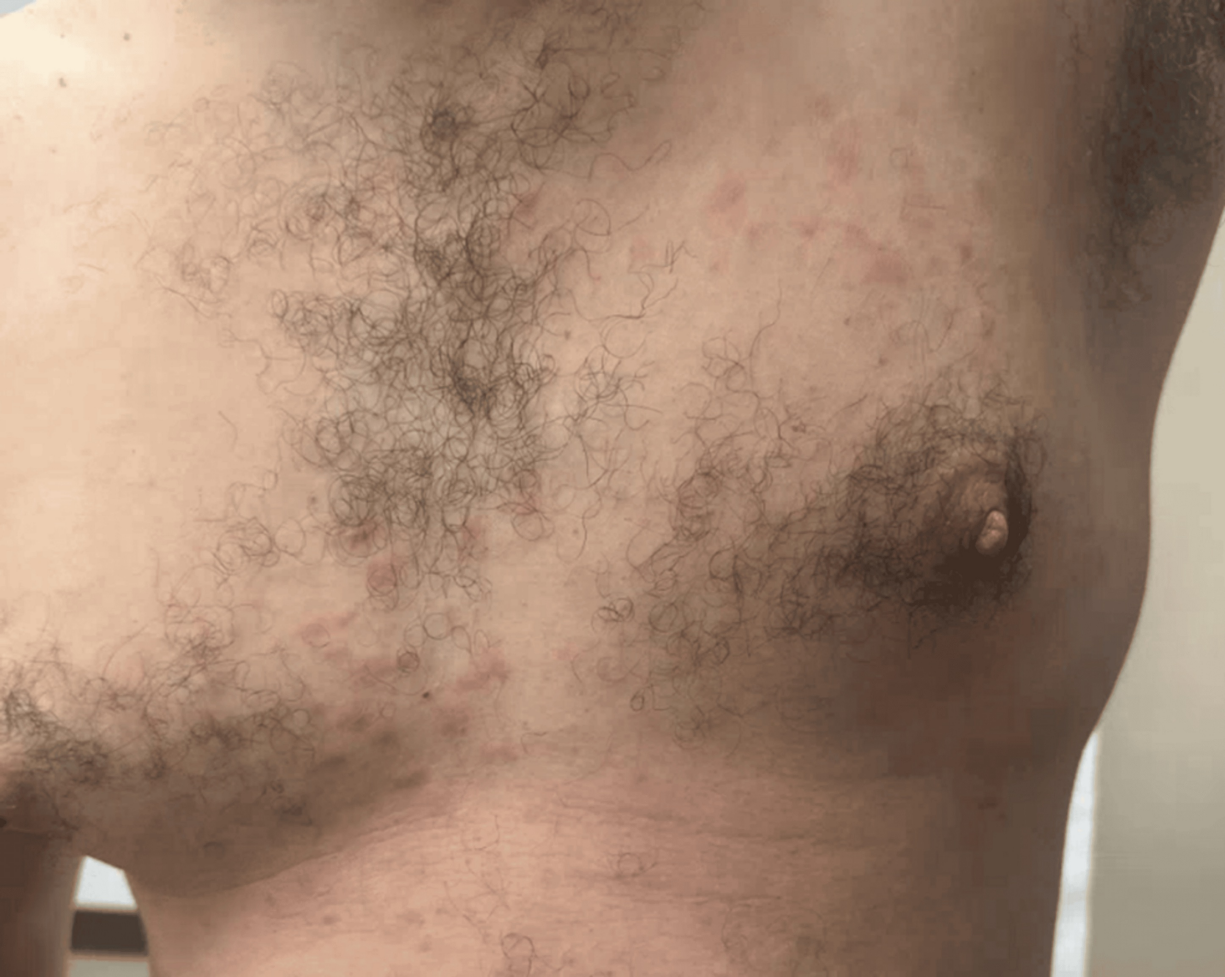
Recurrence of cutaneous eruption following discontinuation of topical corticosteroids The patient's chest shows erythematous papules coalescing into plaques

**Figure 3:**
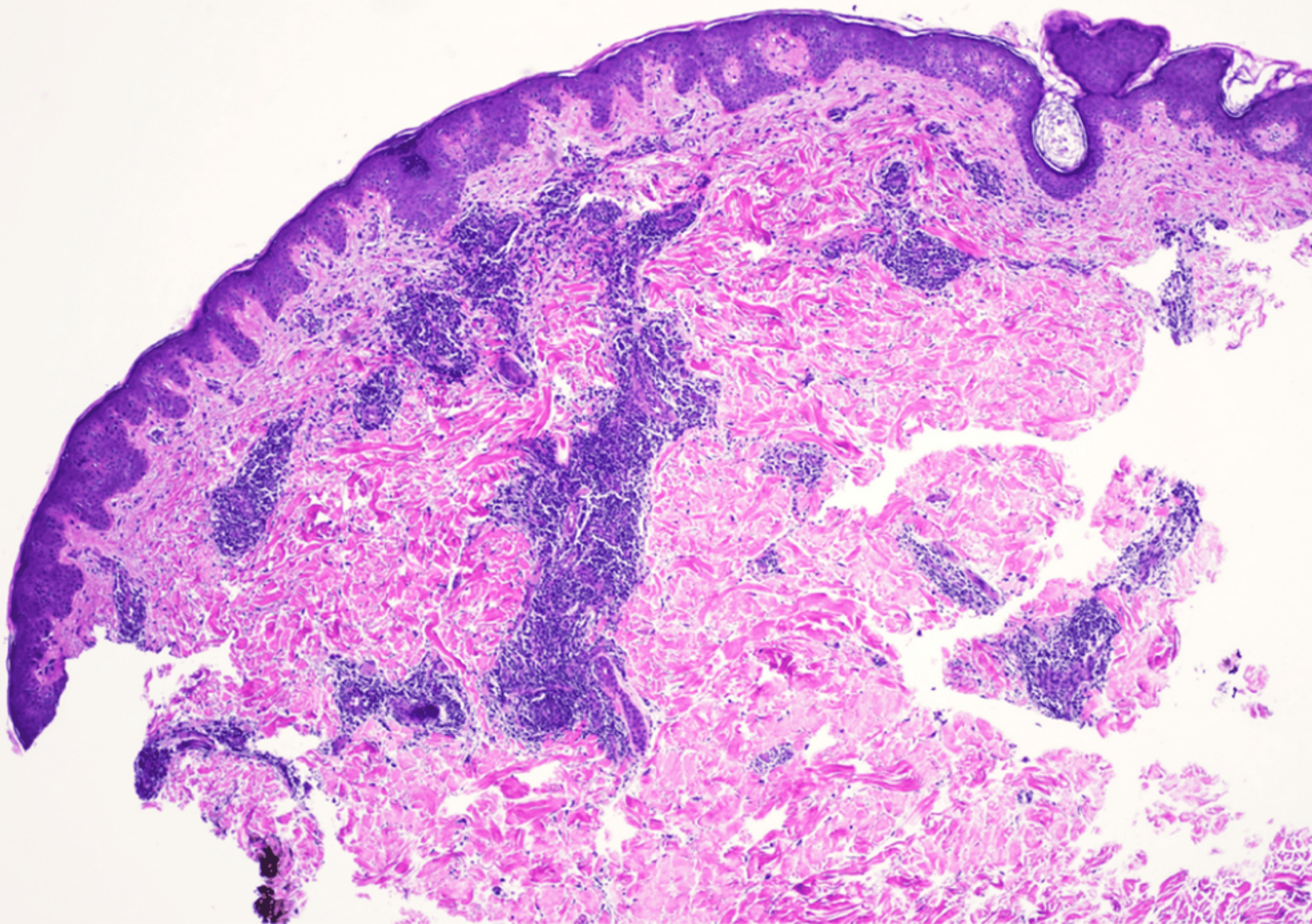
Histopathology consistent with cutaneous lupus erythematosus A punch biopsy stained with hematoxylin and eosin at 4× magnification shows perivascular infiltrates of lymphoid cells, showing variation in nuclear size and staining. They form tight cuffs around blood vessels. The overlying epidermis shows regular acanthosis. Special stains with appropriate controls were prepared. Periodic acid-Schiff stain fails to identify fungal hyphae within the stratum corneum. The infiltrate is CD4-positive and CD8-positive with a ratio of approximately 2:1. CD20 is negative. The pattern is that of a lymphocytic infiltrate of the dermis with dermal mucin. Lupus and other connective tissue disorders would have to be considered

**Figure 4:**
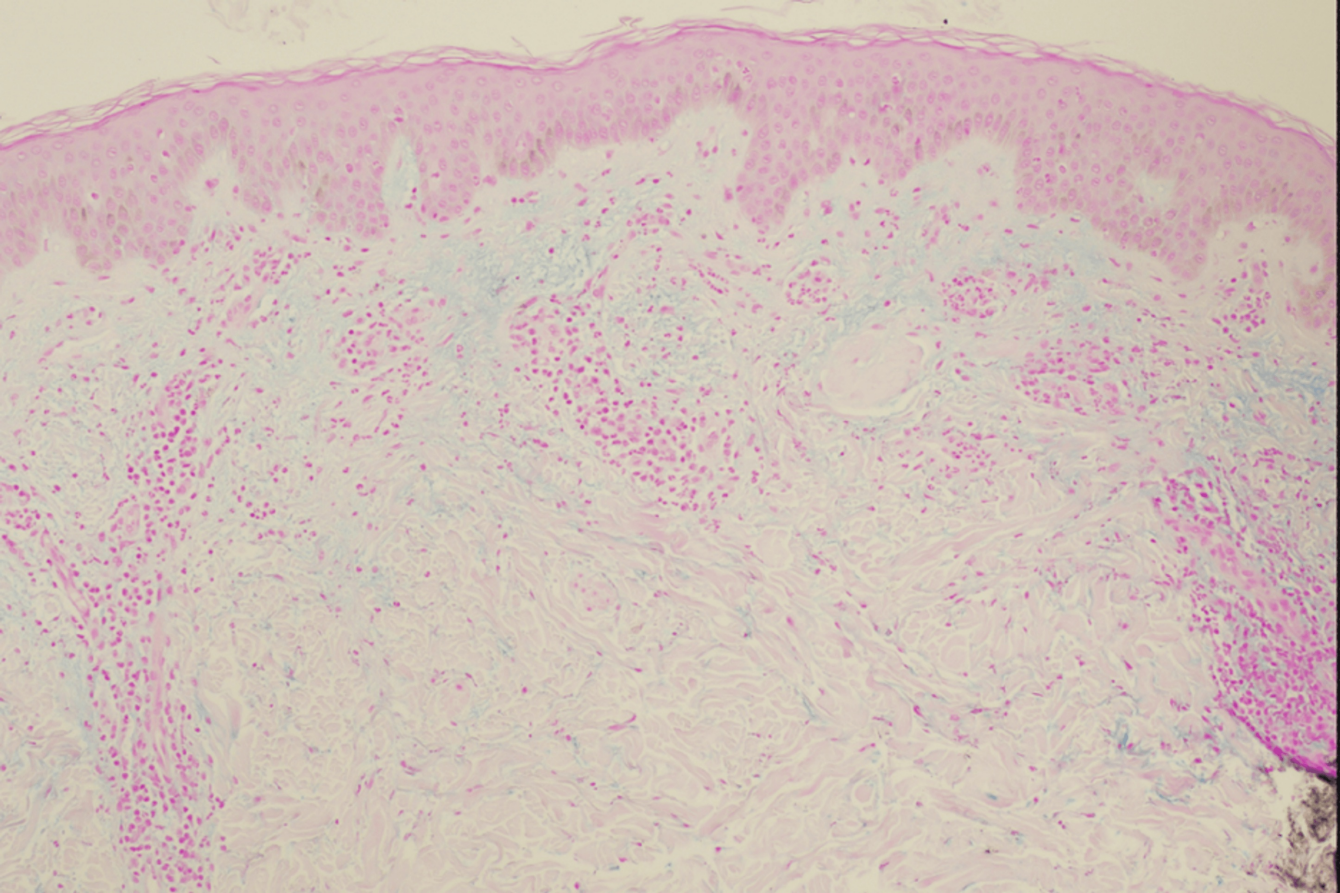
Alcian blue stain demonstrating dermal mucin in cutaneous lupus erythematosus A punch biopsy stained with Alcian blue and colloidal iron at 4× magnification shows prominent mucin deposition within the reticular dermis. The bright blue staining highlights acidic mucopolysaccharides, a characteristic histopathologic feature of cutaneous lupus

By November 2023, the patient experienced a recurrence of his cutaneous eruption after running out of topical steroids (triamcinolone and hydrocortisone) and hydroxyzine (Figures 1, 2). On November 20, 2023, a punch biopsy of the right chest showed a superficial and deep perivascular dermatitis consistent with a possible drug eruption. Hydrochlorothiazide, which he had been taking for hypertension, was discontinued, although the clinical presentation did not fully align with a typical drug-induced process. In January 2024, another flare prompted a second punch biopsy on the back, revealing a lymphocytic infiltrate with dermal mucin suggestive of CLE (Figures 3, 4). He began hydroxychloroquine 200 mg daily for two weeks, then 200 mg twice daily, with partial improvement in his skin lesions. Despite negative anti-double-stranded DNA, negative or fluctuating antinuclear antibody (ANA) titers, and mixed serologies such as positive anti-ribonucleoprotein (anti-RNP) and anti-smith (anti-Sm) in late 2023, there remained a concern for a lupus spectrum disease.

Over the next few months, the patient reported intermittent urticarial lesions that resolved within 24 hours, often accompanied by transient joint swelling in the wrists, knees, and fingers. A third biopsy from the left shoulder on April 1, 2024, demonstrated lichenoid and neutrophilic dermatitis with perivascular and periadnexal inflammation, dermal mucin deposits, and negative direct immunofluorescence (DIF). While some histologic features were compatible with lupus, the findings also supported a picture of neutrophilic urticarial dermatosis (NUD), possibly an SLE, associated neutrophilic process. Because the patient’s clinical picture was atypical for a single unifying diagnosis, additional considerations included Sweet’s syndrome, an early evolving vasculitis such as urticarial urticarial vasculitis, or a drug-induced reaction. Nevertheless, his cutaneous findings and partial response to Plaquenil led to a working diagnosis of tumid lupus with overlapping or atypical features.

By June 2024, he reported only intermittent flares every few days, and laboratory monitoring was generally reassuring, aside from mild normocytic anemia and chronic leukocytosis. He remained on hydroxychloroquine 200 mg twice daily, and his cutaneous disease continued to show improvement with fewer flares.

In September 2024, the patient presented for routine follow-up and reported that his skin remained clear with no recurrences. He continued to tolerate hydroxychloroquine without adverse effects and denied any systemic symptoms. Physical examination showed no active skin lesions and no joint swelling or tenderness. A review of prior histopathological findings highlighted the persistent pattern of perivascular inflammation, lymphocytes, neutrophils, and dermal mucin, consistent with a lupus spectrum or neutrophilic urticarial process. Given that his cutaneous disease had not flared for several months, the plan was to continue hydroxychloroquine 200 mg twice daily until one year of therapy (approximately February 2025), at which point tapering might be considered if he remained asymptomatic.

Currently, the patient’s clinical course is most consistent with tumid lupus or a lupus-related neutrophilic dermatosis responsive to hydroxychloroquine. He remains under close dermatologic and rheumatologic surveillance with ongoing medication monitoring, ready for treatment adjustment should new flares or systemic features arise.

## Discussion

This patient’s clinical course underscores the inherent challenges in diagnosing and managing lupus spectrum skin conditions. His cutaneous eruption was initially presumed to be atopic dermatitis, supported by a transient improvement with dupilumab yet complicated by fever of unknown origin and diarrhea. However, there is also the possibility that dupilumab contributed to or exacerbated an underlying autoimmune process through a Th1-driven mechanism. Although a direct causal relationship remains unproven, case reports have described lupus-like phenomena arising in temporal association with dupilumab initiation, suggesting that immune modulation by this agent may unmask or worsen subclinical autoimmune conditions [7].

Subsequent workup revealed histopathologic features consistent with a drug eruption, followed by findings indicative of cutaneous lupus. These sequentially evolving tissue biopsy results, ranging from superficial and deep perivascular dermatitis to dermal mucin deposition with mixed neutrophilic and lymphocytic infiltrates, reflect the dynamic nature of his disease process.

One important diagnostic consideration was the presence of “urticarial-like” transient lesions and arthralgias, which prompted evaluation for NUD and other autoimmune or autoinflammatory syndromes (e.g., Sweet’s syndrome, early vasculitis). Negative DIF further complicated the picture, as a positive DIF often supports lupus in ambiguous cases. Nonetheless, the patient’s partial and sustained improvement on hydroxychloroquine strongly suggested an underlying lupus spectrum disorder or rheumatologic process [5,6].

Tumid lupus, a subset of chronic cutaneous lupus, is typically characterized by indurated, nonscarring, photosensitive lesions with marked dermal mucin deposition on histology [10,11]. While the patient’s presentation did not follow a classic pattern, his ultimate clinical course, improvement of lesions with ongoing hydroxychloroquine therapy, supports this working diagnosis or a related lupus spectrum dermatosis. Additionally, his variable serologic profile (fluctuating ANA, positive anti-RNP, and anti-Sm at different time points) may reflect an evolving or incomplete systemic process. The absence of a clear rheumatologic diagnosis, coupled with negative workups for malignancy and infectious etiologies, further supports a cutaneous-limited or borderline systemic lupus entity.

Overall, the patient’s experience exemplifies how dermatologic and systemic investigations must be integrated over time to accurately define and manage complex lupus spectrum conditions. Regular skin examinations, histopathologic reassessment, and serologic surveillance are crucial for patients whose disease manifestations and serologies do not fit neatly into conventional diagnostic frameworks. As in this case, hydroxychloroquine can be a valuable cornerstone of therapy, allowing for the stabilization of cutaneous disease even in the face of ambiguous or overlapping diagnoses. Continued multidisciplinary collaboration with rheumatology is essential to detect any emerging systemic involvement and to guide long-term management decisions.

## Conclusions

This case illustrates the diagnostic and therapeutic complexities of lupus spectrum dermatoses, particularly when initial presentations mimic other inflammatory skin conditions such as atopic dermatitis. The temporal association of dupilumab initiation with fever, gastrointestinal symptoms, and evolving histopathologic findings suggests that immune modulation may have unmasked or exacerbated an underlying autoimmune process. Although serologic results remained inconsistent, partial and sustained improvement with hydroxychloroquine further supported a lupus spectrum diagnosis. Given these challenges, ongoing clinicopathologic correlation, multidisciplinary collaboration, and close follow-up are critical for refining the diagnosis and guiding effective treatment in similar complex dermatologic presentations.
